# Faster Evolving Primate Genes Are More Likely to Duplicate

**DOI:** 10.1093/molbev/msx270

**Published:** 2017-11-07

**Authors:** Áine N O’Toole, Laurence D Hurst, Aoife McLysaght

**Affiliations:** 1 Department of Genetics, Smurfit Institute of Genetics, Trinity College Dublin, Dublin, Ireland; 2 The Milner Centre for Evolution, Department of Biology and Biochemistry, University of Bath, Bath, Somerset, United Kingdom

**Keywords:** gene duplication, evolutionary rates, duplicability, primates, evolutionary constraint

## Abstract

An attractive and long-standing hypothesis regarding the evolution of genes after duplication posits that the duplication event creates new evolutionary possibilities by releasing a copy of the gene from constraint. Apparent support was found in numerous analyses, particularly, the observation of higher rates of evolution in duplicated as compared with singleton genes. Could it, instead, be that more duplicable genes (owing to mutation, fixation, or retention biases) are intrinsically faster evolving? To uncouple the measurement of rates of evolution from the determination of duplicate or singleton status, we measure the rates of evolution in singleton genes in outgroup primate lineages but classify these genes as to whether they have duplicated or not in a crown group of great apes. We find that rates of evolution are higher in duplicable genes prior to the duplication event. In part this is owing to a negative correlation between coding sequence length and rate of evolution, coupled with a bias toward smaller genes being more duplicable. The effect is masked by difference in expression rate between duplicable genes and singletons. Additionally, in contradiction to the classical assumption, we find no convincing evidence for an increase in $d_{N}/d_{S}$ after duplication, nor for rate asymmetry between duplicates. We conclude that high rates of evolution of duplicated genes are not solely a consequence of the duplication event, but are rather a predictor of duplicability. These results are consistent with a model in which successful gene duplication events in mammals are skewed toward events of minimal phenotypic impact.

## Introduction

It is commonly reported that recently duplicated genes have higher rates of evolution than singleton genes (Lynch and Conery 2000; Van de Peer et al. 2001; Pegueroles et al. 2013). This is often interpreted as evidence that a period of genetic redundancy following gene duplication creates a temporary relaxation of functional constraint, thus permitting faster evolution (Lynch and Conery 2000; Jordan et al. 2004). However, it is not obvious that genetic redundancy causes a relaxation of constraint on coding sequences—particularly, when one considers the likelihood of nonsynonymous substitutions having a dominant negative effect—and cases of accelerated evolution may reflect positive selection rather than a relaxation of purifying selection (Hughes 1994). Indeed, positive selection after gene duplication has been associated with functional innovation in many instances. One clear example is seen in adaptation to a new diet where a new digestive enzyme arose by duplication and divergence from its paralog with all amino acid changes occurring in one copy of the pair (Zhang et al. 2002). Specific amino acid changes after gene duplication have also been associated with changes in ligand specificity of corticosteroids (Arterbery et al. 2011). An alternative scenario, involving positive selection after gene duplication, arises when there is conflict between separate functions of a singleton gene such that neither function can be improved without compromising the other. This restriction of the singleton gene to be a “jack of all trades, master of none” is released by gene duplication which provides an escape from adaptive conflict, as seen in an anthocyanin pathway gene in morning glory plants (Des Marais and Rausher 2008). The latter is an example of a phenomenon first proposed by Hughes and now known as “subfunctionalization,” where functions of a multi-functional ancestral singleton gene get shared out between the daughter paralogs (Hughes 1994). Subfunctionalization may also occur as a passive process involving degenerative mutations, but this is considered more likely for regulatory sequence evolution as opposed to coding sequence evolution (Hughes 1994; Force et al. 1999).

Both the relaxation of selective constraint model and adaptive evolution model assume that the higher rate of sequence evolution of duplicate genes postdates the duplication event and was enabled by the duplication event. There is, however, a rarely considered alternative possibility to explain the correlation between rates of coding sequence evolution and gene copy number. The alternative postulates that the causal arrow runs in the opposite direction and that genes that are intrinsically fast-evolving are more prone to successful duplication. That is to say, if genes with lower sequence constraint (and thus faster rates of evolution) are also under lower copy number constraint, this would explain the differences in rates of evolution between duplicated and nonduplicated genes without invoking a period of relaxed sequence constraint or adaptive evolution. We are concerned with small-scale duplication (SSD) as opposed to whole genome duplication (WGD), which are different in many significant ways. Nonetheless, it is interesting to note that ancestral evolutionary rates influence expression evolution and retention of paralogs following WGD events in teleost fish and in *Xenopus* (Brunet et al. 2006; Sémon and Wolfe 2008).

Under this alternative model, gene duplication is often a symptom of lack of constraint, and we further expect that such weakly constrained genes are easily gained and lost. In this scenario, the greatest chance to observe a duplicate is shortly after it has been created, before it has been lost again. Consistent with this, ancestrally faster-evolving *Xenopus* genes were more likely to be found with one of the WGD-duplicated pair exhibiting reduced expression, (Sémon and Wolfe 2008) possibly *en route* to being lost again. Thus, we predict that such weakly constrained, fast-evolving genes are mostly observed as young duplicates, and that, by contrast, long-term retention of SSD paralogs will be a rarer event perhaps associated with an initial burst of positive selection and ultimately associated with purifying selection.

In this context, being more prone to duplication (higher duplicability) could have several, not mutually exclusive, components. It could mean that the gene in question is more likely to undergo the mutational event generating a copy number change (mutation bias) or that any such mutational event might be more likely to reach fixation (fixation bias). Additionally, it could mean that any fixed event might be more likely to persist over long evolutionary times and so be detected as a duplicated gene (retention bias). Note that these first two biases can only refer to SSD events and not WGD: by definition, WGD includes all genes and there is no fixation step—if the WGD event is successful that implies fixation of all duplicates. Indeed, SSD and WGD are generally not comparable to the extent that the genes commonly duplicated by SSD are usually not ultimately retained following WGD, leading to almost nonoverlapping sets of paralogs generated by the two mechanisms (Guan et al. 2007; Hakes et al. 2007; Makino and McLysaght 2010; Makino et al. 2013). Notably, asymmetric sequence evolution is an observable phenomenon in a sizeable fraction of post-WGD paralogs (that is, one paralog evolves faster than the other) and is consistent with relaxation of sequence constraints for many paralogs (Brunet et al. 2006; Fares et al. 2006; Byrne and Wolfe 2007). However, asymmetric expression evolution, where one WGD paralog has low or no expression in all tissues, is more prevalent in genes with ancestrally higher rates of sequence evolution (Sémon and Wolfe 2008), which may indicate consistently low evolutionary constraints, rather than a change induced by the duplication status.

In the case of SSD, mutational biases are quite possible. For example, if recombination is mechanistically coupled to the generation of new copies, then genes in genomic domains with higher recombination rates might be more prone to the mutational gain of an extra copy (Woods et al. 2013). Indeed, nonessential genes in *Caenorhabditis* tend to reside in genomic domains with higher recombination rates (Woods et al. 2013), and hotspots of segmental duplication exist in the human genome (Mefford and Eichler 2009). Similarly, genes that are highly expressed in germline are more likely to undergo a transmissible retrocopying event (Vinckenbosch et al. 2006). For genes that are highly dosage sensitive, one can similarly envisage that any change of dosage via a new duplication event might be opposed by purifying selection (Rice and McLysaght 2017). This may explain why members of protein complexes tend to be resilient to SSD (Papp et al. 2003). Hence there may well exist a filter enabling faster rates of copy number evolution of genes that are not dosage sensitive (Woods et al. 2013). Assuming dosage insensitive genes to be faster evolving (Hurst and Smith 1999), a relationship between duplicability and faster sequence evolution can be expected. However, if a gene is more easily gained it might also be more easily lost, so the interplay of fixation bias and retention bias may be complex (for review see Zhang and Yang 2015).

The duplicability hypothesis requires that faster sequence evolution is correlated with faster copy number changes. Consistent with this, in yeast, less essential genes are more duplicable (He and Zhang 2006), and in *Caenorhabditis* nonessential genes are more duplicable, more easily lost again, have low expression levels and faster evolution (Woods et al. 2013). However, this Woods et al. analysis did not uncouple rates of sequence evolution from the duplication process itself. To investigate the alternative hypothesis properly, we need to know about intrinsic rates of evolution of genes independent of the duplication event in question.

In order to determine whether observed faster rates of evolution in duplicated genes reflect a postduplication acceleration or instead a persistently higher rate of evolution independent of gene duplication, we seek to measure rates of evolution prior to and independent of any duplication event. Primates were an ideal choice for study organisms due to the high-quality genome sequence and gene annotation available for many species and their inclusion in EnsemblCompara (Vilella et al. 2008). The study organisms selected were gibbon (*Nomascus leucogenys*) and macaque (*Macaca mulatta*). From these two species, we identified genes that are singletons (and were ancestrally singletons). We estimated the rate of evolution of these singletons alone in a pairwise mode. We assume the rate of evolution in this outgroup pair is a defensible proxy for the ancestral rate. Duplicability was assessed based on whether or not orthologs of these singleton genes had duplicated in any of four closely related crown primate species (human, *Homo sapiens*; chimpanzee, *Pan troglodytes*; gorilla, *Gorilla gorilla*; and orangutan, *Pongo abelii*). We then partitioned the data into genes that have or have not duplicated and compared their rates of evolution in the singleton ancestors, thus uncoupling the estimation of the rate of sequence evolution from the process of duplication itself.

We find that genes that duplicated in the great apes (duplicable genes) have higher rates of evolution in their closely related outgroup, thus supporting the alternative model of greater duplicability of fast evolving genes. This study design also has the advantage of being focused on a relatively recent time period, so should reveal the patterns, if any, shortly postduplication rather than much longer term retention biases. We conclude that, at least in primates, faster sequence evolution is correlated with faster rates of copy number alteration. We examine possible covariates that might underpin this result. We find no evidence for a systematic acceleration of sequence evolution postduplication, nor for rate asymmetry between paralogs.

## Results

### Identification of Primate Singleton and Duplicate Genes

Previous studies have applied various different approaches to the detection of duplicate genes, each of which has its caveats. One popular approach is based on all-against-all BLAST searches (Lynch and Conery 2000; Davis and Petrov 2004; Jordan et al. 2004; He and Zhang 2006; Woods et al. 2013). Additionally, gene families—from which duplicate genes may be inferred—can be constructed using gene sequence clustering methods or distance-matrices (Lan and Pritchard 2016). Alternatively, gene family trees can be used to confirm paralogy (Pegueroles et al. 2013; Li et al. 2016). Phylogenetic tree based methods are the gold standard, being integral to the distinction between orthologs and paralogs (Fitch 1970, 2000) but might not be implemented for reasons of computational tractability, though in many cases precomputed gene trees are available (such as from Ensembl; Vilella et al. 2008).

**Fig. 1.**
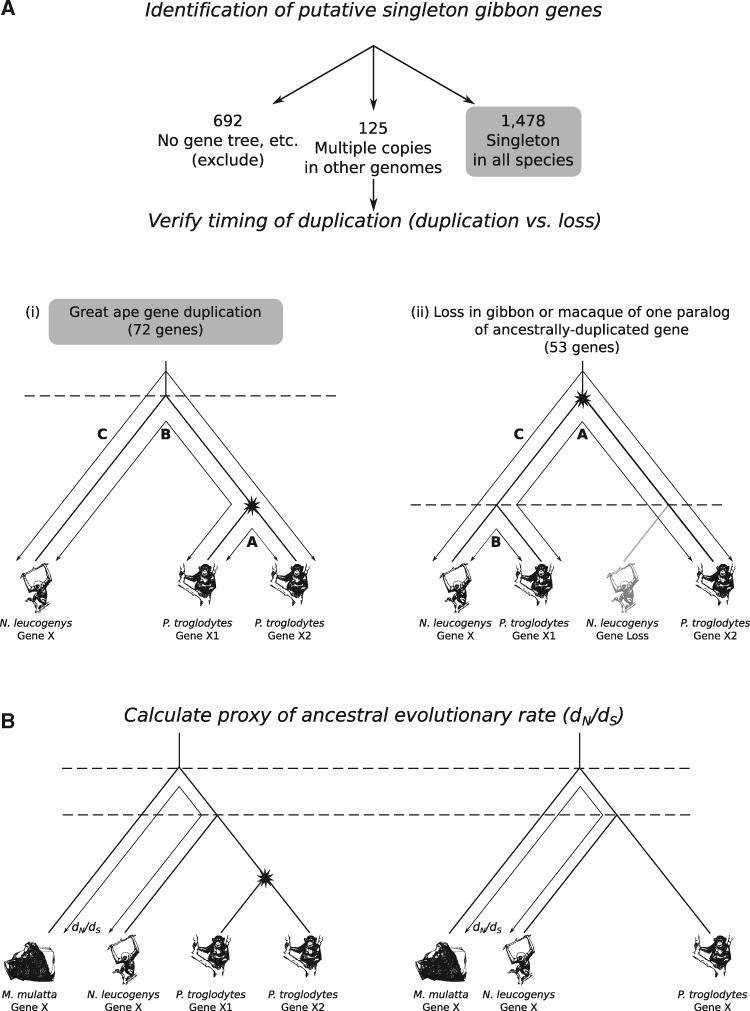
**Project strategy.** We are interested in identifying genes that are singletons in gibbon and macaque, and sorting those into great ape singleton and duplicable genes according to their status in great ape genomes. **(*a*)** Compile list of putative singleton gibbon genes (via an all-against-all BLASTp). For each singleton gene, obtain the corresponding Ensembl gene tree. Confirm the singleton status of gibbon and macaque within the trees. Assess their condition (singleton or nonsingleton) in each of the four related primate species human, chimpanzee, gorilla, and orangutan. This restriction to within the primate lineage minimizes the effects of young versus old duplications. The nonsingleton set of gene trees can arise through gene duplication or gene loss events. The two alternative scenarios are illustrated using chimpanzee (panels *a*—i and ii). If a duplication has occurred in the chimpanzee lineage, the distance, *A*, between the two sister genes will be less than each of the distances to the orthologous gibbon gene (*B* and *C*). However, this relationship will not hold if the nonsingleton arose from a prespeciation duplication event, with a subsequent gene loss in gibbon. This method has been adapted from a similar protocol in He and Zhang (2006). **(*b*)** The rate of evolution ($d_{N}/d_{S}$) in macaque and gibbon is used as a proxy for the ancestral rate of evolution of the primate lineage, independent of any duplication event. Horizontal dashed lines represent speciation events and stars represent gene duplication events.

We performed a systematic search for genes that were singletons at the base of the primate tree and remain singletons in macaque and gibbon, and then classified these as either singleton or duplicable based on their duplication status in the great apes inferred from gene tree analysis (fig. 1*a*). Any ancestral singleton gene that was duplicated in at least one of the great apes was considered as “duplicable.” We employed several stringent quality control steps to account for ancestral duplication and loss, as described in methods, and excluded cases where appropriate. We identified 1,550 genes where we could be confident that they were singletons at the base of the primate tree and remain singletons in macaque and gibbon; of these 1,478 are also singletons in all the great apes examined, and 72 are duplicated in at least one great ape genome (hereafter “singleton” and “duplicable” genes, respectively).

### Duplicable Genes Evolve at Faster Rates than Singletons

The entire set of retained gene trees, whether singleton or duplicable in the great apes, share the status of macaque, gibbon, and the ancestral node as singletons. First, to confirm the previously reported (Lynch and Conery 2000) higher evolutionary rate in duplicated genes (i.e., postduplication) we measured the rate of evolution of the 1,478 singletons and 72 duplicates. We find that the rate of protein evolution of singletons ($d_{N}/d_{S}$ between the human and macaque sequence) is lower than for duplicates (average of $d_{N}/d_{S}$ between the each of the paralogs and the macaque sequence), with medians of 0.25 and 0.38, respectively (Mann–Whitney *U* test $d_{N}/d_{S}$, *W* = 42,468, *P* value = 0.0037; supplementary fig. S2, Supplementary Material online). This is consistent with the findings of previous studies (Lynch and Conery 2000; Van de Peer et al. 2001; Pegueroles et al. 2013) but does not reveal the cause-and-effect relationship. Does duplication cause higher rates of evolution, as predicted by the classic model, or rather, is a faster rate of evolution characteristic of duplicable genes?

As we are interested in investigating the evolutionary rate of singletons and duplicates independently of the potential effects of the duplication event, we measured the amount of evolution (*d_N_*, *d_S_*, and their ratio, $d_{N}/d_{S}$) in the macaque and gibbon lineages and use this as a proxy for the ancestral evolutionary rate. We are aware of only one prior analysis which tests for an evolutionary-rate bias in SSD gene duplicability and which considers the rate of sequence evolution of genes in one lineage (*D. melanogaster* and *A. gambiae*) while defining duplicability by reference to events in a different lineage (*S. cerevisiae* and *C. elegans*). The authors report that duplicable genes evolve slower, not faster, than those that had not duplicated (Davis and Petrov 2004). However, this analysis was not ideal in that it did not control for duplication in the lineage in which evolutionary rates were calculated and the two lineages were very distant (and so gene function need not be conserved and there might be an ascertainment bias toward more essential genes).

Any sequences with values of $d_{S}<0.01$ or $d_{N}/d_{S}>10$ were excluded from our analysis as they can potentially reflect inflated and inaccurate $d_{N}/d_{S}$ values (Yang 2007; Villanueva-Cañas et al. 2013). We confirmed that this exclusion does not significantly affect the results (supplementary table S1, Supplementary Material online). This left 1,543 alignments in total: 70 in the duplicable set and 1,473 in the singleton set. All alignments with $d_{S}>0.3$ (*n* = 62) were visually inspected and were found to have well-aligned codons, thus we concluded that the alignments were reliable.

**Fig. 2.**
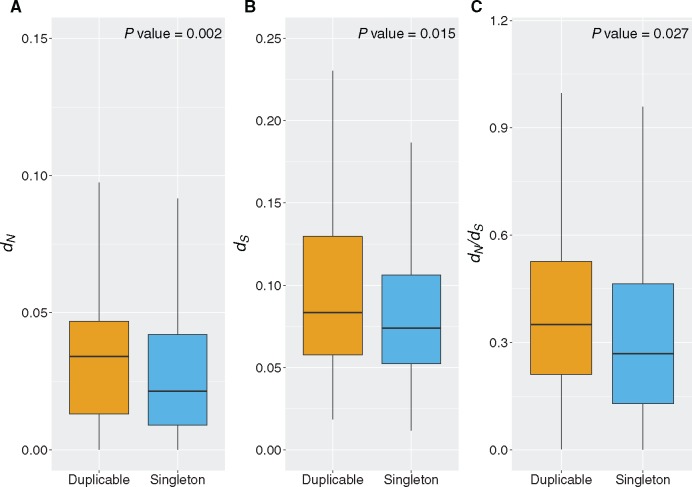
**Boxplots showing. *d_N_*, *d_S_*, and** $d_{N}/d_{S}$ **values for duplicable and singleton gene sets.** All genes have singleton status in macaque and gibbon, and the evolutionary rates were calculated by comparison of macaque-gibbon orthologs. The data are separated into singleton and duplicable genes according to their status in the great apes, with the latter having at least one duplication event in the great apes. Values were compared using the Mann–Whitney *U* test and *P* values are shown above each pair of boxplots.

There are significant differences between singletons and duplicable genes in *d_N_*, *d_S_*, and $d_{N}/d_{S}$ values (Mann–Whitney *U* test; *W* = 40,210, 42,662, 43,521; *P* = 0.002, 0.015, and 0.027, respectively). The duplicable genes consistently show higher rates of synonymous and nonsynonymous substitution even though these measurements are based on macaque-gibbon sequence comparison where the genes have not experienced duplication (fig. 2). Median values for *d_N_*, *d_S_*, and $d_{N}/d_{S}$ are 0.04 and 0.02; 0.1 and 0.08; and 0.36 and 0.27, for duplicable and singleton gene trees, respectively. In other words, at least a component of the elevated rates of sequence evolution of duplicated genes is independent of their duplication status. This is suggestive of generalized lower constraints on duplicable genes, observed both as greater duplicability and faster sequence evolution. This is consistent with previous studies that found greater duplicability of less essential genes (He and Zhang 2006; Woods et al. 2013).

One version of the model of evolution after gene duplication posits that purifying selection should be initially relaxed due to redundancy, but then re-established once the duplicate acquires a new function. Lynch and Conery (2000) compared *d_N_* and *d_S_* in pairs of paralogous genes (i.e., postduplication) and found that whereas $d_{N}\approx d_{S}$ when *d_S_* is low, *d_N_* < *d_S_* when *d_S_* is high. This was interpreted as a signal of an initial relaxation of selective constraint on young duplicates ($d_{N}/d_{S}\approx 1$), followed later by purifying selection and presumed functionality ($d_{N}/d_{S}<1$).

**Fig. 3.**
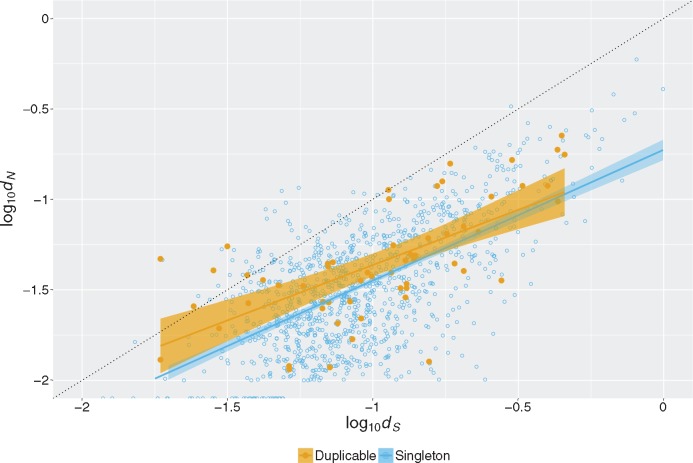
**Greater difference between *d_N_* and *d_S_* at higher *d_S_* for both duplicable and singleton genes.** An xy-scatterplot of $log10d_{N}$ versus $log10d_{S}$ values for duplicable and singleton genes. The black dotted line indicates *d_N_* = *d_S_* (neutral evolution). An ANOVA comparing two models with/without duplication status incorporated as an interaction term implies duplication status does not significantly affect the observed trend (*F* = 1.0876, *P* value = 0.34).

With our data, we have the opportunity to compare *d_N_* and *d_S_* in a similar way, but for all genes independent of duplication events. We find that the slope of *d_N_* predicted by *d_S_* is < 1 (fig. 3). Hence the difference between *d_N_* and *d_S_* increases with higher *d_S_*, even though in this case *d_S_* is not a surrogate for time as all of the genes compared here should have the same divergence time (the macaque-gibbon speciation). Instead, in our analysis, variation in *d_S_* predominantly indicates variation in mutation or fixation rates. That is, we find a similar pattern to Lynch and Conery (2000) independent of duplication status and time. This argues against the prior interpretation supposing that this pattern is a consequence of (and evidence for) duplication-induced alteration of constraints.

### Why Might Singleton Genes Evolve Slower than Duplicable Genes?

#### Expression Level Differences Mask the Difference in $d_{N}/d_{S}$

**Fig. 4.**
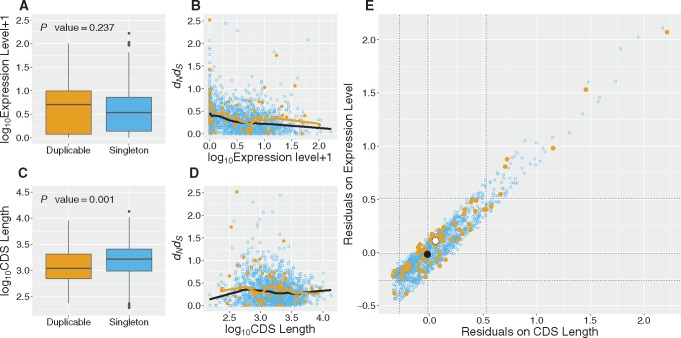
**Rate differences between singleton and duplicable genes are explained by CDS length and expression level differences.** Analysis of 1,473 singletons (blue) and 70 duplicable genes (orange). Boxplot of (*a*) *log*_10_ of expression levels + 1 (log2-transformed RPKM gene expression data) and (*c*) log_10_ of CDS lengths of duplicable and singleton genes. *P* values for the test of difference between the means is shown above the boxplots (*P* values 0.237 and 0.001, respectively, MWU). LOWESS regression fitted for $d_{N}/d_{S}$ on the log_10_ of (*b*) expression levels + 1 and (*d*) CDS length. The regression lines for singletons are shown in black and, for duplicates in orange. (*e*) $d_{N}/d_{S}$ residual space is graphed by plotting the residuals extracted from the two LOWESS regressions. The median residual value of the duplicable set is indicated by a large white point and the singleton set by a large black point. The dotted lines from each axis indicate the medians and 95 limits for the respective sets of residuals.

Why might genes that subsequently duplicate have higher rates of protein evolution and higher $d_{N}/d_{S}$. Gene expression level has been shown to be a strong and universal predictor of evolutionary rate such that expression levels of slowly evolving genes are significantly higher than of fast evolving genes in yeast (Pál et al. 2001; Drummond et al. 2005) and mammals (Wang et al. 2011). Should genes prone to duplication have lower expression levels, then their faster evolution may in part be explained by such an effect. Thus we investigated whether the differences in evolutionary rates between duplicable and singleton genes are explained by differences in expression levels. We sourced expression level data in macaque from Brawand et al. (2011). We confirmed that the rate of evolution negatively correlates with expression level (Spearman’s ρ=−0.35, *P* value = <0.0001, for entire data set). We find no significant difference in expression level between duplicable and singleton genes (fig. 4*a*: Mann–Whitney *U*, *W* = 47,246, *P* value = 0.237), although duplicable genes have if anything slightly higher expression levels: mean of log_10_ (expression + 1) for duplicables: 0.66 ±0.065 SEM, mean for singletons =0.56 ± 0.01 SEM.

That duplicates have if anything a higher expression level suggests that the underlying difference between singletons and duplicates in rates of evolution is in part being masked. To address this possibility we fitted a LOWESS regression for $d_{N}/d_{S}$ on the log_10_ of macaque expression data (fig. 4*b*). When we examine the residuals of this regression line, we find that the higher $d_{N}/d_{S}$ in duplicates than singletons becomes more significant (Mann–Whitney *U* test of the LOWESS expression residuals: *W* = 41,909, *P* value = 0.008, previously *P* value = 0.027), consistent with expression level differences masking the rate differences between singletons and duplicates. To ask whether the reduction in *P* value after residuals correction is itself significant, we performed a nonparametric Monte Carlo simulation in which the expression values were randomized, the LOWESS and subsequent residuals recalculated and the difference between duplicate and singleton genes in residuals recalculated. In 10,000 simulants we find no simulant with as low a *P* value as the actual residuals, strongly supporting the view that the slightly raised expression level of the duplicate genes masks a more profound difference between duplicable and singleton genes in their $d_{N}/d_{S}$ values.

#### CDS Size Differences Explain Some of the Difference in $d_{N}/d_{S}$

A priori we might expect that gene length might also be an important parameter to examine, not because the correlation between gene length and $d_{N}/d_{S}$ is especially strong (Nembaware et al. 2002), but length differences between duplicable and singleton genes might be expected for many reasons: retroposition might favor shorter genes to duplicate; and longer genes might be more costly to duplicate if there are translation costs. Larger proteins (hence longer genes) do indeed tend to be more deleterious upon overexpression (Ma et al. 2010).

Similar to a prior report (Nembaware et al. 2002), we find a negative correlation between $d_{N}/d_{S}$ and coding sequence (CDS) length, more modest that the expression level effect (Spearman’s ρ=−0.1, *P* value <0.0001). In addition, we find that singletons are longer than duplicable genes (fig. 4*c*: Mann–Whitney *U* test, *W* = 63,530, *P* value = 0.001, mean log_10_ CDS length in bp of duplicates: 3.06 ±0.04 SEM, mean for singletons = 3.2 ± 0.008 SEM).

To determine whether this length difference significantly explains some of the difference between duplicable and singleton genes in their rates of evolution, comparable to the above, we fitted a LOWESS regression for $d_{N}/d_{S}$ onto the log_10_ of CDS length (fig. 4*d*). Comparing the residuals for regression of CDS-length suggests that some of the difference between the duplicable and singleton gene tree values has been accounted for as the *P* value has increased from 0.027 to 0.046 (Mann–Whitney *U*, *W* = 44,272). To determine whether the increase in *P* value is itself significant we again perform a nonparametric Monte Carlo simulation, generating residuals from the plot of $d_{N}/d_{S}$ against randomized log_10_ CDS length, and again compare residuals for the duplicable and singleton genes via a Mann–Whitney *U* test. In 10,000 simulations only 43 had a *P* value from this Mann–Whitney *U* test as high or higher than that observed with the real data, indicating that some significant (*P* value = 0.004) fraction of the difference between duplicates and singletons in $d_{N}/d_{S}$ is explained by the fact that singletons are longer and longer genes tend to evolve slower.

For completeness, we also considered genomic length. While the correlation between genomic length and $d_{N}/d_{S}$ is even weaker than that for CDS length (Pearson’s *r* = −0.06, *P* value = 0.02), we observe no difference in genomic length between singleton and duplicable genes (Mann–Whitney *U*, *W* = 35,118, *P* value = 0.0848). As above, we fitted a LOWESS regression for $d_{N}/d_{S}$ on log_10_ of genomic length and find that, upon comparing residuals from this LOWESS regression between singleton and duplicable genes, the *P* value has increased from 0.027 to 0.031. After simulation testing as described earlier we find that this increase in *P* value is not significant and genomic length does not explain a significant portion of the variation (randomization test, *P* value = 0.097).

We can consider the combined impact of expression and CDS length differences on $d_{N}/d_{S}$ by comparing the residuals from the above analyses (fig. 4*e*). A Kernel Density Estimation of the 2D spread of residual values for duplicate and singleton genes indicates the difference in $d_{N}/d_{S}$ has been accounted for by the two covariates (*P* value = 0.32). The difference in $d_{N}/d_{S}$ values between duplicable and singleton genes is thus accounted for in some part by the differences in CDS length and expression level.

A further possible cause of the difference in $d_{N}/d_{S}$ between duplicable and singletons is a potential difference in G and C content affecting codon usage bias, mutagenicity, and $d_{N}/d_{S}$. To address this we have compared GC3 (mean of the macaque and gibbon value for each gene) between duplicable and singleton genes. While $d_{N}/d_{S}$ correlates with GC3 content (*ρ*=−0.18, *P* < 0.001), GC3 is not different between duplicable and singleton genes: Mann–Whitney *U* test, *P* value = 0.495, mean GC3 percentage for duplicables = 57.5 ±1.84, for singletons = 56.2, ±0.41. The *P* value for Mann–Whitney *U* test on the residuals of $d_{N}/d_{S}$ predicted by GC3 is very close to that observed prior to the residuals test (for $d_{N}/d_{S}$ before residuals test *P *=* *0.027, after *P *=* *0.0263). This is not significantly different from the null expectation (randomization test, *P*= 0.31). We conclude that differences in GC3 do not explain differences in $d_{N}/d_{S}$ between duplicable and singleton genes.

### No Evidence for Relaxation of Evolutionary Constraints following Duplication

Even if faster evolving genes are more duplicable than slowly evolving genes, it is still possible that duplication causes a period of relaxed functional constraint or positive selection. A common hallmark of adaptive evolution or relaxed purifying selection is taken to be rate asymmetry between the paralogs. To test for rate asymmetry, we considered a likelihood framework considering a model in which paralogs are forced to have the same rate compared with a comparable model where they are permitted to have different rates. Given that the duplications within the primate lineage are recent, so as not to bias against cases where one of two duplicates has an especially low rate of evolution, in consideration of asymmetry, we remove the requirement for *d_S_* > 0.01. Prior to multitest correction, in only 6 cases out of 52 gene trees does the alternative model permitting asymmetric evolution of paralogs perform better than the null model, and following FDR correction we find no evidence that a model permitting duplicates to have different rates performs significantly better than a model in which duplicate genes are forced to have the same rate (supplementary table S2, Supplementary Material online). We also applied Fisher’s method for combining *P* values and observed no evidence that duplicates evolve at asymmetric rates postduplication when considered *en masse* ($\chi^{2}$ statistic = 82.9, d.f.=104, *P* = 0.94).

The key signature of relaxed selection or positive selection of duplicates is an increase in $d_{N}/d_{S}$ postduplication. Given no evidence for asymmetric evolution between paralogs, we compared two models, the first enforcing a single evolutionary rate across all branches of the primate tree and a second model allowing the rate to vary postduplication. Of the 39 cases, 25 have a higher rate postduplication and the other 14 have a lower rate. This is not significantly different from a 50: 50 split (binomial test, *P* =0.11). In only four cases does the model permitting a distinct evolutionary rate postduplication perform significantly better than the null model (at raw *P* < 0.05) and none of these cases is robust to FDR correction.

As instances where the duplicates evolve slower than the ancestral rate provide no support for the hypothesis of rate acceleration, to be generous to the acceleration model, we considered *en masse* the 25 instances of faster rates. Applying Fisher’s method, we combined the *P* values from our likelihood analysis and observe no convincing evidence of significant net rate acceleration postduplication ($\chi^{2}$ statistic = 63.37, d.f.=50, *P* = 0.097). That this is true even when excluding all examples that *prima facie* argue against the model, we conclude that we see no evidence for increased $d_{N}/d_{S}$ in any given gene or when considered *en masse*.

## Discussion

Gene duplication is a common occurrence in eukaryotic evolution. Many models invoke a period of relaxed selection due to redundancy following duplication as the context for the evolution of novel functions in paralogs. Alternatively adaptive evolution may be enabled by gene duplication. In both cases the supposition is that the faster evolution of duplicated genes is evidence for such effects and, by assumption, must occur post the duplication event. However, we find no evidence that paralogs evolve at different rates postduplication nor for asymmetrical rates of evolution of the duplicates. Rather, our results support an alternative model wherein genes with faster rates of evolution, presumably reflecting lower functional constraints, are inherently more duplicable. These results are consistent with a model in which the set of duplicable genes is less constrained in terms of both sequence evolution and copy number evolution. The underlying cause of the increased duplicability, whether it be owing to mutation bias, fixation bias or retention bias, remains to be discerned. Given the short amount of time we are examining in this study (i.e., great-ape-specific duplication events), in our opinion retention biases are less important than in other studies examining longer periods of evolutionary time (Davis and Petrov 2004). To ascertain the magnitude of any effect would require forward simulations.

These results provide a consistent framework for considering several previous findings: that less essential genes are more duplicable in yeasts (He and Zhang 2006); that young genes are less essential than older genes (Chen et al. 2012); that young duplicates are short-lived and are less essential than older duplicates (Woods et al. 2013); and that duplicability is a consistent characteristic of a subset of genes, even across vast evolutionary distances (Li et al. 2016). All of these results make sense under the model where a subset of the genome is only weakly constrained in terms of both sequence evolution and copy number. Duplicable genes are fast evolving and have “easy come, easy go” paralogs, frequently fixed and lost again (presumably by drift in both instances).

In this context, the generalizability of our results invites scrutiny. That small genes evolve faster and are more duplicable explains some of the trend for duplicable genes to be faster evolving. Given this, the trend that we have identified need not apply to all modes of duplication. We have examined duplicates that are not the result of whole genome duplication events. While we do not know why small genes are overrepresented in the set of genes that successfully duplicated, many possible causes of the size effect on duplicability are unlikely to apply equally to WGD events. For example, genes with a small CDS might be more duplicable because they are more likely to fully integrate as retrocopied genes or be less costly when overexpressed. If so, then WGD and SSD will have different dynamics.

Furthermore, it is unclear whether our results are expected to extend to small scale duplicates in other lineages. Indeed, one might conjecture that in the large-bodied primates that we considered, with small effective population sizes, the dynamics are more likely to be dominated by neutral and nearly neutral forces. Whether the same trend for faster evolution to be coupled with duplicability exists in lineages with more efficient selection owing to larger effective population sizes, is worthy of scrutiny. One prior analysis (Davis and Petrov 2004) did examine the coupling of evolutionary rate and duplicability in invertebrates and yeasts and reported a result opposite to ours, that is, that duplicable genes evolve slower, not faster, than those that had not duplicated. How might we understand the difference in conclusions?

One possibility is, as we conjecture above, a difference in the strength of selection. Before accepting this possibility, we sought to replicate the prior analysis using updated data. The bulk of the evolutionary sequence rate analysis in the Davis and Petrov study was reported for all sequences without considering only those that are singletons in the outgroup. The authors do parenthetically mention this control but do not elaborate on it. We have attempted to replicate the analysis using only singletons in the relevant lineage (see Supplementary Material online). However, we find that no genes meet the criteria of being singletons in the outgroup and duplicate in the ingroup, thus we could proceed no further. This could possibly be explained if a subset of genes is inherently duplicable, and thus unlikely to be duplicated in one lineage while remaining unduplicated over the large evolutionary times encapsulated by this study setup (Li et al. 2016). Our analysis additionally estimates evolutionary rates in lineages in close phylogenetic proximity to the duplication event. Owing to this our analysis probably has a reduced filter of any retention bias, as the time for gene loss is much more restricted than in the prior study. It would be valuable to repeat our mode of analysis on closely related species with large population sizes (e.g., yeast, flies). It would be similarly valuable to see if duplicates from whole genome duplication events behave differently to those resulting from small scale duplication events.

Our data support the view that duplicable genes are less important, and their loss is less consequential, whether or not they are duplicated (He and Zhang 2006; Woods et al. 2013). Our study tests the model that duplicable genes are a less constrained subset of the genome ab initio, and shows, at the very least, that the observed faster rates of evolution of duplicate genes is not necessarily a consequence of events that occur after the duplication event. 

## Materials and Methods

### Data

Whole proteome data were downloaded from Ensembl ftp for both gibbon and macaque (release 83) and protein and nucleotide sequences for all primate study species (human, chimp, gorilla, orangutan, gibbon, and macaque) were collected using the Ensembl REST API (Cock et al. 2009; Yates et al. 2015, version 4.6).

Macaque log2-transformed reads per kilobase per million (RPKM) gene expression data were obtained from the RNA Seq transcriptome data in Brawand et al. (2011). The median expression level across all six available tissues for each gene was obtained and matched to the genes in our analysis using Ensembl Gene IDs. CDS-length values from macaque are used in all cases with the data sourced from Ensembl. Genomic length information was calculated from genomic start and stop positions downloaded from Ensembl BioMart (Kinsella et al. 2011).

### Definition of Singleton and Duplicable Genes

We defined singleton and duplicable genes within the great apes as individual ancestral genes that had either remained unduplicated or been duplicated at least once, respectively. In order to uncouple duplication status from evolutionary rate measurement we also required that all genes in this study remain singletons in gibbon and macaque, where evolutionary rates will be measured. An initial list of 2,961 candidate singleton gibbon genes was defined as genes with only self-hits in an intraspecific all-against-all BLASTp (*E* value threshold = 0.1) using the longest protein for each gene. Gene trees were obtained from Ensembl Compara, the generation of which is described in Vilella et al. (2008), and these were pruned using ETE3, a python framework for phylogenetic analysis (Huerta-Cepas et al. 2016), to include only species of interest (Gibbon, macaque, orangutan, gorilla, human, and chimp). We employed several quality control filters using Python to verify the singleton status at the base of this pruned tree and to exclude ancestral duplication followed by loss in gibbon or macaque (graphical summary in supplementary fig. S1, Supplementary Material online). Of the 2,961 putative singleton gibbon genes, 692 were excluded because they either lacked a gene tree, a macaque ortholog, or lacked any identifiable orthologs in any other genome (these could be annotation artifacts, novel genes, or very rapidly evolving genes); 1,926 gene trees had a single gibbon and a single macaque gene; and 343 trees had more than one homolog in gibbon and/or macaque (potentially ancestrally duplicated).

The 343 gene trees with multiple macaque or gibbon homologs need further examination to assess whether or not they can be included in this analysis. Where the tree topology indicated that the duplication predated the primate lineage such that there were subtrees being made up of a single macaque and single gibbon gene with orthologs in the great apes, these were split and retained as distinct gene family trees (supplementary fig. S1, Supplementary Material online).

We identified 1,478 gene trees containing a single macaque and gibbon gene and also a single homolog in orangutan, gorilla, chimp, and human and were thus considered singleton gene trees. In the set of gene trees where at least one of the four great ape species had more than one gene within the gene tree, the observed gene counts could have arisen via a gene duplication event in the great apes, or gene loss events in macaque and gibbon. As this study is specifically interested in identifying great-ape-specific gene duplications, it was necessary to rule out gene loss as an explanation for the gene counts. For the set of 125 gene trees of the nonsingletons (more than one copy in at least one of the great apes) we evaluated whether the gene copy number was due to a recent great-ape-specific duplication or to an ancestral duplication with loss in some lineages. We used genetic distance between paralogs to distinguish ancestral and recent duplication events in a protocol similar to He and Zhang (2006) where the paralogs are considered to be created by a recent duplication event when the genetic distance between the paralogs is less than the genetic distance from either one to the gibbon gene. If the distance between, say, two chimpanzee paralogs (*A*) is less than the distance between the gibbon ortholog and each of the sister chimpanzee genes (denoted *B* and *C*, respectively), we rule out ancestral duplication with lineage-specific loss and infer that the duplication event occurred more recently than the speciation event (that is, within the time period of interest here; illustrated in fig. 1*a*). Genetic distances were obtained from the Ensembl gene trees (Vilella et al. 2008). If more than two paralogs per species were present, the two most closely related paralogs were considered. The method is potentially confounded by interparalog gene conversion. However, we do not think this is a substantial issue and moreover, under these circumstances, the macaque-gibbon distance would appear as a very obvious outlier and we see no evidence for this.

Of the 125 gene trees with only one ortholog in macaque and gibbon and multiple orthologs in some great ape genomes, we cannot rule out ancestral duplication followed by gene loss in macaque and gibbon for 53 gene trees. The remaining 72 gene trees include great ape gene duplication events.

### Test for Differing Rates of Evolution between Singleton and Duplicate Genes

For each of the 1,478 singleton gene trees and the 72 duplicate gene trees, the protein sequences of the gibbon, macaque, orangutan, gorilla, chimpanzee, and human genes were aligned using MUSCLE (Edgar 2004) and then converted to nucleotide alignments using Translator-x (Abascal et al. 2010). Lists of duplicate and singleton families are provided as Supplementary Material online. To test whether duplicate genes and singleton genes evolve at different rates, as has been described previously (Lynch and Conery 2000; Van de Peer et al. 2001; Pegueroles et al. 2013), we extracted the human and macaque sequences from the multiple sequence alignments and estimated $d_{N}/d_{S}$ using the codeml module of PAML 4.8, set to runmode =−2 for pairwise rate calculation, CodonFreq = 2, with all other parameters as default. For each duplicate gene within a gene tree, we calculate the pairwise rate with the macaque sequence and then take a mean rate for the gene tree. A graphical summary of PAML usage is shown in supplementary figure S3, Supplementary Material online.

### Measurement of Proxy-Ancestral Evolutionary Rate

To calculate proxy ancestral rates of evolution (fig. 1*b* and supplementary fig. S3, Supplementary Material online), we extracted the aligned singleton gibbon and macaque sequences from the multiple sequence alignment and used them in the calculation of $d_{N}/d_{S}$. By extracting the alignment of just these genes, we prevent PAML from allowing evolutionary rate calculations dependent on the evolution of the genes after duplication to interfere with or otherwise affect the inference of the rate when they are singletons. Any effects of gene conversion postduplication, for example, cannot then interfere with ancestral rate estimation. We estimated *d_N_*, *d_S_*, and $d_{N}/d_{S}$ as above. Gene trees with $d_{N}/d_{S}$ values > 10 or *d_S_* values < 0.01 were excluded from further analysis (as per Villanueva-Cañas et al. 2013), as low *d_S_* values may artificially inflate $d_{N}/d_{S}$.

### Model Selection

In order to investigate whether the regression lines of *d_N_* versus *d_S_* in figure 3 are significantly different between singleton and duplicable measurements, model selection was performed using Akaike information criterion (AIC) values, which are a measure of the relative quality of a given statistical model taking into account the goodness-of-fit and complexity of a given model. We compared a null model of $d_{N}∼d_{S}$ and an alternative model incorporating an interaction term for *d_S_* and duplication status. The AIC values suggest that incorporating this interaction term for *d_S_* and duplication status does not produce a superior model ($d_{N}∼d_{S}$, AIC = −6,614.2; $d_{N}∼d_{S}$, + status + *d_S_*: status, AIC = −6,612.3. ANOVA comparing the two models, *F* = 1.0876, *P* value = 0.34). This suggests that this trend is true for both singletons and duplicable genes.

### Examination of Possible Explanatory Variables

In order to determine whether a possible covariate of both duplicate/singleton classification and evolutionary rate might mask or explain differences in evolutionary rate, we employed a nonparametric regression approach, with significance determined by nonparametric Monte Carlo simulations. Briefly, for the variable in question (we consider log_10_ expression + 1, gene length measured as log_10_ CDS length, genomic length, and G + C content at the third position of codons, GC3), we construct the LOWESS regression of this *x* variable against $d_{N}/d_{S}$. We employed the *lowess* function in R with f = 0.3, with the fit established via the *approxfun* function. From the best fit regression line we calculate the deviation of each data point in $d_{N}/d_{S}$ from the expected value given the regression line and the value of the *x* variable (values above the line having positive residuals, those below having negative residuals). These residual values we then compare between the duplicable and singleton genes via Mann–Whitney *U* test. We checked the resilience of results to employing the alternative *loess* function in R and found no qualitative differences in results.

We evaluate the significance in the change in *P* value between the residual corrected and the uncorrected values (i.e., the *P* value of the Mann–Whitney *U* test comparing $d_{N}/d_{S}$ between duplicated and singleton genes), via randomization. Here, we randomly reassign the values of the *x* variable without replacement and recalculate the LOWESS regression and the residuals in the same manner as above, using the randomized *x* variable as the predictor. We then recalculate the difference between duplicated and singleton genes as before, extracting the reported *P* value. Repeating this process 10,000 times we ask how often we observe a *P* value as extreme or more extreme as that observed when the LOWESS is performed with unrandomized data. The *P* value for this test is the number of observations as extreme or more extreme divided by the number of simulations. The median value of the *P* value in the simulants should be very nearly the same as the *P* value of Mann–Whitney *U* test comparing singletons and duplicates with no covariate correction, as the regression against random data should have an average slope of zero. With a slope of zero, the rank order of the residual values would be the same as the rank order of the observed values and hence the partitioning into duplicated and singleton genes should give the same pairs of rank orderings and hence the same *P* value. We confirmed that median *P* value of the simulants was indeed very similar to that of the uncorrected analysis.

### Testing for Asymmetric Evolution of Paralogs

In order to test whether there was significant asymmetric evolution between paralogs postduplication we ran the branch-model of codeml (runmode = 0, model = 2, See supplementary fig. S3, Supplementary Material online) for each gene tree for each duplicated species, comparing two models: first with paralogs forced to have the same $d_{N}/d_{S}$ value and second an alternative model with the paralogs allowed to have distinct evolutionary rates. The first, null model allowed a background rate (*ω*_0_) and then all paralogs for a given species were given a rate *ω*_1_ (two parameter model). The alternative model allowed each paralog to have a distinct rate. In the case of two paralogs in a species, the model allows a background rate (*ω*_0_), as before, and two postduplication rates (*ω*_1_ and *ω*_2_). If more than two paralogs exist for a given species per gene tree, additional rates are allowed (*ω*_3_, etc.). We excluded gene trees with $d_{N}/d_{S}$ > 10, leaving 52 duplication events. The Log-likelihood values and numbers of parameters were extracted from the PAML output files for each model and the LRT test statistic, the number of degrees of freedom (DOF) between the two models and *P* values were calculated using Python and R. Supplementary table S2, Supplementary Material online, gives details of DOF and *P* values, pre- and postfalse discovery rate (FDR) correction. We also implemented Fisher’s method for combining *P* values using the metap package for R (Dewey 2017).

### Testing for Accelerated Evolution Postduplication

Akin to the asymmetry analysis, we compared two codeml models to test for rate acceleration (supplementary fig. S2, Supplementary Material online). The first null model implemented a single evolutionary rate (*ω*_0_) for all branches in the primate tree (runmode = 0, model = 0). The second model allowed two evolutionary rates within a gene tree: the background rate *ω*_0_ and a distinct postduplication rate *ω*_1_ for duplicated genes within a species (runmode = 0, model = 2). We excluded gene trees with *d_S_* < 0.01 and $d_{N}/d_{S}$ > 10 (as recommended in Villanueva-Cañas et al. 2013). Due to the recency of many duplications, after applying this filter 39 duplication events remained in the analysis. As before, the log-likelihood from the PAML output was used to calculate LRT test statistics and *P* values (See supplementary table S3, Supplementary Material online, for details). We identified cases where the postduplication rate of evolution was higher than the background rate and, as above, implemented Fisher’s method for combining *P* values (Dewey 2017).

### Data Handling

Unless otherwise stated, statistical tests and plots were performed and created using R, sm, and ggplot2 (Bowman and Azzalini 2014; Wickham 2016). The kernel density estimation (KDE) test is a nonparametric test for testing whether two 2D sets of data are the same. It was run using the *ks* (kernel-smoothing) package for R (Duong 2017).

## Supplementary Material

Supplementary data are available at *Molecular Biology and Evolution* online.

## Supplementary Material

Supplementary Data
