# Supplementary material for: Faster Evolving Primate Genes Are More Likely to Duplicate
Source: Mol Biol Evol. 2017 Nov 7;35(1):107–18. doi: 10.1093/molbev/msx270 (PMC5850500; doi:10.1093/molbev/msx270)
Supplement: Supplementary Data [file msx270_supp.zip › supporting_information.pdf]

Supporting Information

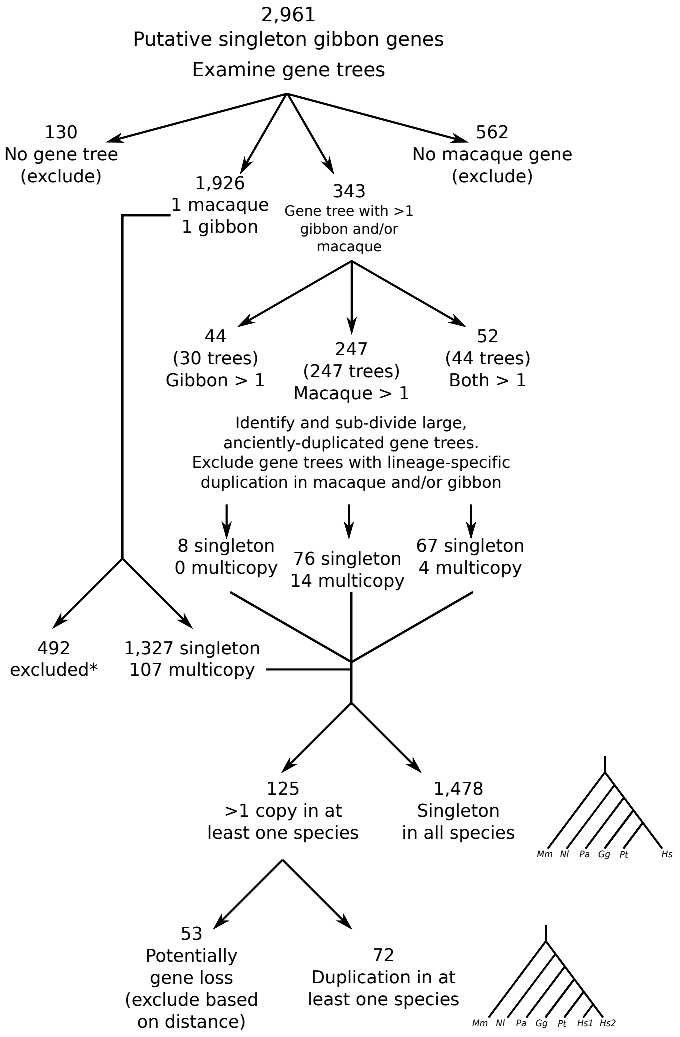

Figure S 1: **Gene tree filtering work-flow.** This figure describes the filtering steps carried out on the Ensembl gene trees. The initial 2,961 putative singleton gibbon genes were identified by an all-*vs*-all BLASTP. Putative singletons were defined as those genes with no significant hit (E-value$<$ 0.1) other than a self-hit. 343 gene trees had multiple gibbon and/or macaque genes, which could potentially have been due to an ancestral duplication. These trees were subdivided to contain only the more recent gene history and were assessed for singleton or non-singleton status. Singleton gene trees, by definition, have a singleton gene in macaque and gibbon and in each of the four great ape species examined. Gene trees were considered multicopy (non-singleton) if they had one gibbon and one macaque gene, but more than one gene in at least one of the great ape species. *492 gene trees were excluded from further analysis for either gene loss events in trees with no duplications or the outermost species of the gene tree not being macaque and gibbon.

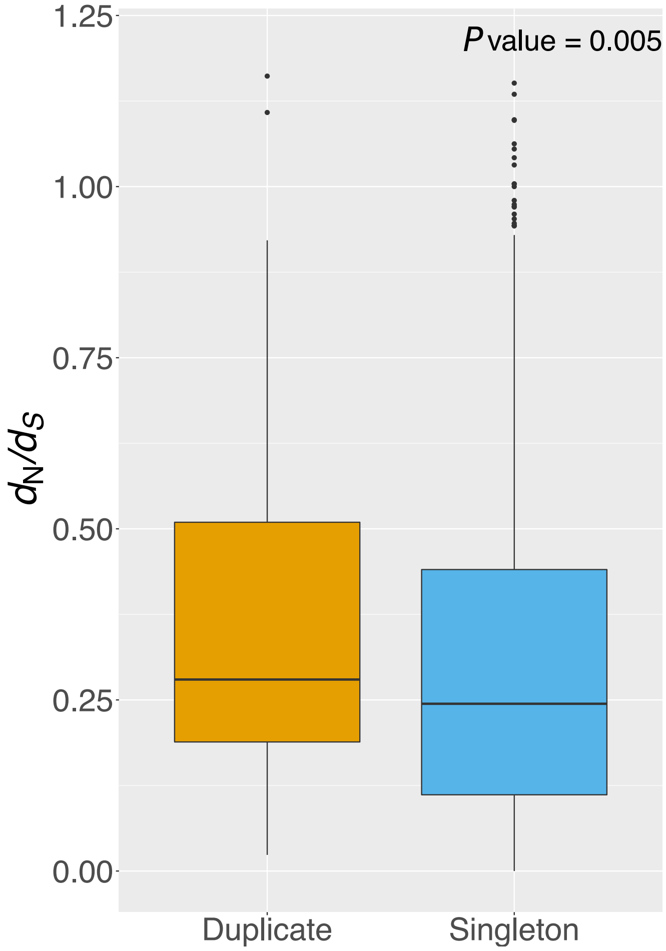

Figure S 2: **Boxplots of d_N/d_S Values for Duplicate and Singleton Genes.** For singletons d_N/d_S was calculated from alignments between macaque and human. For duplicate genes the rate of evolution was measured post-duplication (i.e., not an ancestral rate of evolution): a mean value of d_N/d_S was obtained from pairwise rate calculations between macaque and each of the paralogs. Measurements of d_N/d_S values > 10 and $d_S < 0.001$ were excluded, leaving 71 duplicate and 1,474 singleton genes. We observe a significant difference in d_N/d_S between duplicates and singletons (Median values: duplicate = 0.37. singleton = 0.25; Mann-Whitney U test: W = 42094, P value = 0.005).

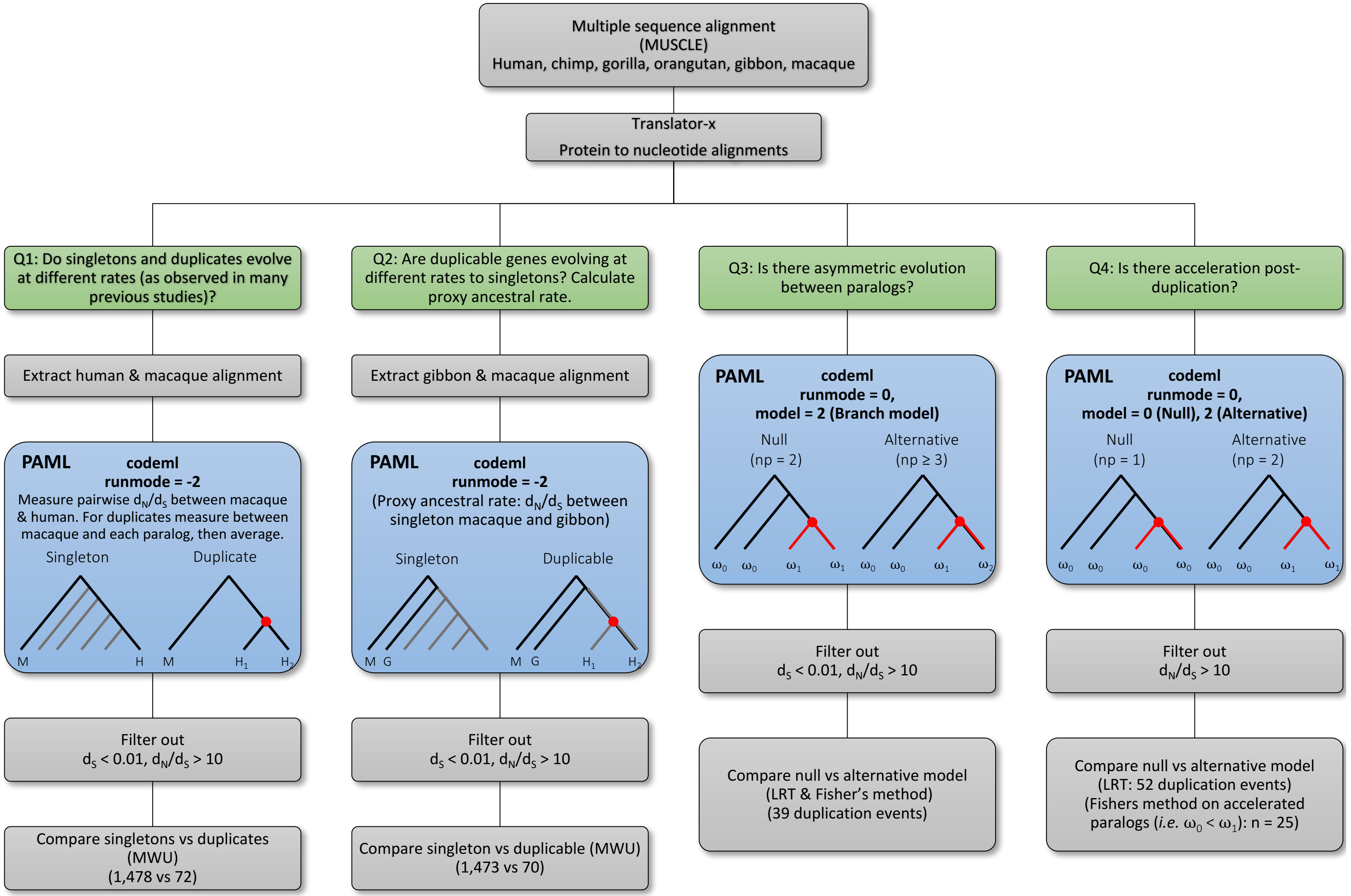

Figure S 3: **PAML usage summary.** This figure describes the various tests we performed using codeml. ω = evolutionary rate, Red circle = duplication event, G = Gibbon, H = Human, M = Macaque, H_1 and H_2 = paralogous human genes, np = number of parameters, MWU = Mann Whitney U, LRT = Likelihood ratio test.

Table S 1: **Effects of filtering on comparisons of Duplicable *vs* Singleton genes**. Filtering consisted of first excluding outliers (i.e. $d_N/d_S > 10$) and then also excluding cases of d_S <0.01 as small d_S values can cause artificial inflation of d_N/d_S. P values from Mann-Whitney U tests.

Comparison	No filtering		Outliers removed ($d_N/d_S > 10$)		Removed outliers and $d_S < 0.01$	
	n	P value	n	P value	n	P value
d_N	72 *vs* 1,475	0.0006	70 *vs* 1,474	0.002	70 *vs* 1,473	0.002
d_S		0.016		0.014		0.015
$\mathrm{d}_N/\mathrm{d}_S$		0.028		0.027		0.027

Table S 2: **Testing for asymmetric evolution between paralogs.**

**Macaque gene**	**Species**	**Null** lnL	**Alt.** lnL	**Test Statistic**	**DOF**	P **value**	**FDR**	d_S
ENSMMUP00000008147	ENSPPYP	-389.86	-389.86	0.0	1	1.0	1.0	0.0127
ENSMMUP00000032191	ENSP000	-736.63	-736.63	0.0	1	1.0	1.0	0.0429
ENSMMUP00000025803	ENSPTRP	-321.85	-321.85	0.0	1	1.0	1.0	0.0527
ENSMMUP00000025803	ENSP000	-324.11	-324.11	0.0	1	1.0	1.0	0.0037
ENSMMUP00000020910	ENSPPYP	-2384.99	-2384.84	0.2817	1	0.5956	1.0	0.0325
ENSMMUP00000018260	ENSPPYP	-346.87	-344.72	4.2902	1	0.0383	0.6213	0.0591
ENSMMUP00000036966	ENSPTRP	-3211.94	-3208.94	6.0078	1	0.0142	0.5207	0.053
ENSMMUP00000003670	ENSP000	-1651.48	-1651.48	0.0	1	1.0	1.0	0.004
ENSMMUP00000021341	ENSGGOP	-2275.02	-2274.38	1.275	1	0.2588	1.0	0.1378
ENSMMUP00000016573	ENSPPYP	-1462.62	-1460.62	4.0046	13	0.9911	1.0	0.0695
ENSMMUP00000024262	ENSPPYP	-1444.41	-1444.41	0.0014	1	0.9702	1.0	0.0236
ENSMMUP00000035028	ENSP000	-802.21	-802.21	0.0	1	1.0	1.0	0.0094
ENSMMUP00000019005	ENSPPYP	-1710.46	-1709.8	1.3329	1	0.2483	1.0	0.0524
ENSMMUP00000013815	ENSPPYP	-460.73	-460.73	-0.0001	1	1.0	1.0	0.0125
ENSMMUP00000004761	ENSPTRP	-2482.42	-2481.3	2.222	1	0.1361	1.0	0.0104
ENSMMUP00000020596	ENSP000	-496.88	-496.88	0.0	1	1.0	1.0	0.056
ENSMMUP00000010825	ENSPTRP	-104.61	-104.61	0.0	1	1.0	1.0	0.3523
ENSMMUP00000019904	ENSPPYP	-789.63	-789.63	0.0	1	1.0	1.0	0.0069
ENSMMUP00000027733	ENSGGOP	-1890.24	-1890.24	0.0	1	1.0	1.0	0.0026
ENSMMUP00000027733	ENSP000	-1889.17	-1889.09	0.1675	1	0.6823	1.0	0.0153
ENSMMUP00000009450	ENSGGOP	-487.16	-486.5	1.315	1	0.2515	1.0	0.1032
ENSMMUP00000039451	ENSP000	-1415.11	-1415.11	0.0	1	1.0	1.0	0.0054
ENSMMUP00000025344	ENSPTRP	-542.96	-542.96	-0.0001	1	1.0	1.0	0.0212
ENSMMUP00000021553	ENSGGOP	-2208.1	-2208.1	0.0	1	1.0	1.0	0.008
ENSMMUP00000020679	ENSP000	-413.38	-413.38	0.0084	1	0.927	1.0	8e-04
ENSMMUP00000027517	ENSP000	-1620.68	-1620.68	0.0	1	1.0	1.0	0.0158
ENSMMUP00000036478	ENSPPYP	-508.84	-506.05	5.5801	1	0.0182	0.5207	0.0195
ENSMMUP00000036478	ENSPTRP	-508.13	-508.13	0.0	1	1.0	1.0	0.0093
ENSMMUP00000025237	ENSGGOP	-211.7	-211.7	0.0	1	1.0	1.0	19.097
ENSMMUP00000025237	ENSPTRP	-210.47	-210.47	0.0	2	1.0	1.0	0.6076
ENSMMUP00000025237	ENSP000	-211.64	-211.64	0.0	2	1.0	1.0	0.1251
ENSMMUP00000032228	ENSPPYP	-454.15	-453.62	1.0549	1	0.3044	1.0	0.0374
ENSMMUP00000032228	ENSPTRP	-455.2	-453.28	3.8225	1	0.0506	0.6213	0.0181
ENSMMUP00000019182	ENSGGOP	-2465.54	-2465.51	0.066	1	0.7973	1.0	0.0635
ENSMMUP00000019182	ENSPTRP	-2465.56	-2464.92	1.289	1	0.2562	1.0	0.0813
ENSMMUP00000021569	ENSPPYP	-4458.09	-4454.84	6.503	1	0.0108	0.5207	0.0596

Table S 2: **Testing for asymmetric evolution between paralogs**.

**Macaque gene**	**Species**	**Null** lnL	**Alt.** lnL	**Test Statistic**	**DOF**	$\boldsymbol{P}$ **value**	**FDR**	d_S
ENSMMUP00000013830	ENSPTRP	-1762.91	-1762.91	0.0	1	1.0	1.0	0.0114
ENSMMUP00000029593	ENSPPYP	-471.82	-471.65	0.3306	1	0.5653	1.0	0.0233
ENSMMUP00000007980	ENSGGOP	-729.8	-728.69	2.2231	1	0.136	1.0	0.0061
ENSMMUP00000008937	ENSPPYP	-511.74	-511.41	0.6638	1	0.4152	1.0	0.0143
ENSMMUP00000008937	ENSP000	-511.93	-511.93	-0.0001	1	1.0	1.0	0.0073
ENSMMUP00000006099	ENSPPYP	-583.23	-583.23	0.0	1	1.0	1.0	0.0202
ENSMMUP00000023683	ENSPPYP	-547.9	-545.87	4.0534	1	0.0441	0.6213	0.0177
ENSMMUP00000023683	ENSPTRP	-548.32	-548.32	0.0	1	1.0	1.0	0.047
ENSMMUP00000023683	ENSP000	-547.24	-546.73	1.0226	1	0.3119	1.0	0.0661
ENSMMUP00000033283	ENSP000	-765.95	-766.12	-0.3353	1	1.0	1.0	3e-04
ENSMMUP00000014322	ENSGGOP	-535.21	-535.21	0.0	1	1.0	1.0	0.0555
ENSMMUP00000014322	ENSPTRP	-535.99	-535.99	0.0001	1	0.992	1.0	0.0074
ENSMMUP00000014322	ENSP000	-534.2	-534.2	0.0001	3	1.0	1.0	0.0028
ENSMMUP00000039131	ENSPTRP	-460.66	-457.3	6.7237	2	0.0347	0.6213	0.0109
ENSMMUP00000039131	ENSP000	-460.63	-458.02	5.2062	7	0.6348	1.0	0.0028
ENSMMUP00000040521	ENSPPYP	-1514.86	-1514.48	0.7449	1	0.3881	1.0	0.0207

Table S 3: **Testing model of distinct d_N/d_S post duplication**. Null model = single d_N/d_S for all primate sequences, alternative model = distinct d_N/d_S post-duplication.

**Macaque gene**	**Species**	**Null** lnL	**Alt.** lnL	**Test Statistic**	**DOF**	***P* value**	**FDR**	d_S	d_N/d_S	**Post-duplication** d_N/d_S	$\Delta d_N/d_S$
ENSMMUP00000008147	ENSPPYP	-389.95	-389.86	0.178	1	0.6731	1.0	0.0127	0.03194	0.001	-0.031
ENSMMUP00000032191	ENSP000	-737.69	-736.63	2.1098	1	0.1464	0.5708	0.0429	0.63543	0.6756	0.04
ENSMMUP00000025803	ENSPTRP	-324.68	-321.85	5.6625	1	0.0173	0.2376	0.0527	0.28793	0.7256	0.438
ENSMMUP00000020910	ENSPPYP	-2386.41	-2384.99	2.8589	1	0.0909	0.5708	0.0325	0.56722	0.6819	0.115
ENSMMUP00000018260	ENSPPYP	-346.87	-346.87	0.0122	1	0.912	1.0	0.0591	0.07962	0.1473	0.068
ENSMMUP00000036966	ENSPTRP	-3211.95	-3211.94	0.0121	1	0.9124	1.0	0.053	0.42889	0.5042	0.075
ENSMMUP00000021341	ENSGGOP	-2275.67	-2275.02	1.3054	1	0.2532	0.8039	0.1378	0.73905	0.6733	-0.066
ENSMMUP00000016573	ENSPPYP	-1462.78	-1462.62	0.3094	1	0.578	1.0	0.0695	0.52633	0.2945	-0.232
ENSMMUP00000016573	ENSGGOP	-1462.78	-1462.65	0.2592	1	0.6107	1.0	0.1148	0.52633	0.707	0.181
ENSMMUP00000016573	ENSPTRP	-1462.78	-1462.68	0.1965	1	0.6576	1.0	0.0777	0.52633	0.9167	0.39
ENSMMUP00000024262	ENSPPYP	-1447.19	-1444.41	5.5691	1	0.0183	0.2376	0.0236	0.35906	0.3932	0.034
ENSMMUP00000019005	ENSPPYP	-1710.56	-1710.46	0.1984	1	0.656	1.0	0.0524	0.75979	0.921	0.161
ENSMMUP00000013815	ENSPPYP	-461.19	-460.73	0.9139	1	0.3391	0.8254	0.0125	0.18227	0.001	-0.181
ENSMMUP00000004761	ENSPTRP	-2482.99	-2482.42	1.1521	1	0.2831	0.8039	0.0104	0.34015	0.865	0.525
ENSMMUP00000020596	ENSP000	-497.21	-496.88	0.6761	1	0.4109	0.8904	0.056	0.47074	0.7351	0.264
ENSMMUP00000010825	ENSPTRP	-104.61	-104.61	0.0	1	1.0	1.0	0.3523	0.19308	0.5421	0.349
ENSMMUP00000027733	ENSP000	-1891.24	-1889.17	4.1475	1	0.0417	0.4065	0.0153	0.24464	0.5026	0.258
ENSMMUP00000009450	ENSGGOP	-487.17	-487.16	0.018	1	0.8933	1.0	0.1032	0.21518	0.1061	-0.109
ENSMMUP00000025344	ENSPTRP	-544.52	-542.96	3.119	1	0.0774	0.5708	0.0212	1.28653	0.001	-1.286
ENSMMUP00000027517	ENSP000	-1624.28	-1620.68	7.2021	1	0.0073	0.2376	0.0158	0.58975	1.0477	0.458
ENSMMUP00000036478	ENSPPYP	-508.84	-508.84	0.0	1	1.0	1.0	0.0195	0.53087	0.5323	0.001
ENSMMUP00000025237	ENSGGOP	-211.73	-211.7	0.0643	1	0.7998	1.0	19.097	0.17368	0.0501	-0.124

Table S 3: **Testing model of distinct d_N/d_S post duplication**. Null model = single d_N/d_S for all primate sequences, alternative model = distinct d_N/d_S post-duplication.

**Macaque gene**	**Species**	**Null** lnL	**Alt.** lnL	**Test Statistic**	**DOF**	***P* value**	**FDR**	d_S	d_N/d_S	**Post-duplication** d_N/d_S	$\Delta d_N/d_S$
ENSMMUP00000025237	ENSPTRP	-211.73	-210.47	2.5138	1	0.1129	0.5708	0.6076	0.17368	0.4322	0.259
ENSMMUP00000025237	ENSP000	-211.73	-211.64	0.18	1	0.6714	1.0	0.1251	0.17368	0.5384	0.365
ENSMMUP00000032228	ENSPPYP	-455.21	-454.15	2.1259	1	0.1448	0.5708	0.0374	0.73198	0.1494	-0.583
ENSMMUP00000032228	ENSPTRP	-455.21	-455.2	0.0239	1	0.8771	1.0	0.0181	0.73198	0.6469	-0.085
ENSMMUP00000019182	ENSGGOP	-2465.57	-2465.54	0.0659	1	0.7974	1.0	0.0635	0.32879	0.2333	-0.095
ENSMMUP00000019182	ENSPTRP	-2465.57	-2465.56	0.0166	1	0.8975	1.0	0.0813	0.32879	0.166	-0.163
ENSMMUP00000021569	ENSPPYP	-4458.09	-4458.09	0.0101	1	0.9199	1.0	0.0596	0.32555	0.3531	0.028
ENSMMUP00000013830	ENSPTRP	-1762.91	-1762.91	0.0034	1	0.9535	1.0	0.0114	0.63656	1.3742	0.738
ENSMMUP00000029593	ENSPPYP	-471.85	-471.82	0.0601	1	0.8063	1.0	0.0233	0.82629	0.8027	-0.024
ENSMMUP00000008937	ENSPPYP	-511.97	-511.74	0.4518	1	0.5015	1.0	0.0143	0.57073	1.2403	0.67
ENSMMUP00000006099	ENSPPYP	-583.79	-583.23	1.1263	1	0.2886	0.8039	0.0202	0.12219	0.001	-0.121
ENSMMUP00000023683	ENSPPYP	-548.32	-547.9	0.8387	1	0.3598	0.8254	0.0177	1.07014	1.7972	0.727
ENSMMUP00000023683	ENSPTRP	-548.32	-548.32	0.0001	1	0.992	1.0	0.047	1.07014	1.6427	0.573
ENSMMUP00000023683	ENSP000	-548.32	-547.24	2.1594	1	0.1417	0.5708	0.0661	1.07014	1.0508	-0.019
ENSMMUP00000014322	ENSGGOP	-535.99	-535.21	1.5577	1	0.212	0.7516	0.0555	0.64883	0.8958	0.247
ENSMMUP00000039131	ENSPTRP	-460.77	-460.66	0.2273	1	0.6335	1.0	0.0109	1.11654	1.8021	0.686
ENSMMUP00000040521	ENSPPYP	-1515.33	-1514.86	0.938	1	0.3328	0.8254	0.0207	0.56277	0.9104	0.348

S1 Replication of Davis & Petrov (2004)

The Davis and Petrov (2004) reciprocal-best-hit (RBH) method of defining gene duplications was used in our attempted replication of their results: Reciprocal whole-proteome all-against-all BLASTp searches (E-value threshold = 1) were performed on the genomes of the two study organisms, *S. cerevisiae* and *C. elegans*. The version of BLAST used was ncbi-blast-2.2.29+. Singleton genes were defined as those genes without a significant hit (i.e. lacking a hit with an E-value < 0.1), other than a self-hit. Duplicate genes were defined as RBHs with an E-value of less than 10^{-10} in both directions that could be aligned over 60% of the length of the shorter sequence. Similar BLASTp searches were carried out between outgroup species (*D. melanogaster* and *A. gambiae*). Orthologs were defined as RBHs between the two organisms under the same E-value threshold as for duplicates. For both duplicate genes in an RBH pair within a study organism, each gene of that pair had to hit the same gene in the outgroup species. Unlike the main analysis in Davis and Petrov (2004), the criteria we used stipulated that the outgroup species were singletons, i.e., we excluded cases with any outgroup-specific duplications. In this way, duplication events were isolated, independent to duplication events. Davis and Petrov (2004) do extend their criteria to require this in a later section, however few details are given. Counts of genes from the replication of Davis and Petrov (2004) are shown in Table S 4. As can be seen in the table, the use of updated genome builds and BLASTp versions, and incorporating the singleton outgroup species requirement, has altered the counts of genes such that no genes that meet the Davis and Petrov (2004) 'duplicate' criteria to perform an analysis. That is, there were no genes that were duplicated in *C. elegans* and *S. cerevisiae* and also unduplicated in the outgroups. We interpret this as, given enough time, duplicable genes will duplicate. So when the chosen outgroup divergence is very old, it is impossible to find genes unduplicated in the outgroup and duplicated in the ingroup.

Table S 4: **Attempted replication of Davis & Petrov (2004) gene number counts.** The duplicate genes were defined as intraspecific reciprocal-best-hits with an E-value < 10^{-10}; singleton genes were defined as those genes without an intraspecific hit with an E-value < 0.1. The 'With Orthologs' columns are those genes with detectable orthologs (E-value of < 10^{-10}) in *Drosophila* and *Anopheles*. The 'With Sing Orthologs' columns describe those genes which have also had the singleton status of the orthologs in *Drosophila* and *Anopheles* verified. (Dup = Duplicate, Sing = Singleton, D&P = Davis and Petrov, Rep. = Replication.)

No. of genes/ gene pairs	Dup/ Sing		With Orthologs		With Sing Orthologs	
	D&P	Rep.	D&P	Rep.	D&P	Rep.
C. elegans duplicates	1,919	2,134	263	67	38	0
C. elegans singletons	2,229	4,613	723	743	114	359
S. cerevisiae duplicates	448	562	147	30	29	0
S. cerevisiae singletons	1,990	2,845	364	318	318	156
